# Conduction Delay Learning Model for Unsupervised and Supervised Classification of Spatio-Temporal Spike Patterns

**DOI:** 10.3389/fncom.2017.00104

**Published:** 2017-11-21

**Authors:** Takashi Matsubara

**Affiliations:** Computational Intelligence, Fundamentals of Computational Science, Department of Computational Science, Graduate School of System Informatics, Kobe University, Hyogo, Japan

**Keywords:** spiking neural network, temporal coding, delay learning, activity-dependent myelination, spike timing-dependent plasticity, unsupervised learning

## Abstract

Precise spike timing is considered to play a fundamental role in communications and signal processing in biological neural networks. Understanding the mechanism of spike timing adjustment would deepen our understanding of biological systems and enable advanced engineering applications such as efficient computational architectures. However, the biological mechanisms that adjust and maintain spike timing remain unclear. Existing algorithms adopt a supervised approach, which adjusts the axonal conduction delay and synaptic efficacy until the spike timings approximate the desired timings. This study proposes a spike timing-dependent learning model that adjusts the axonal conduction delay and synaptic efficacy in both unsupervised and supervised manners. The proposed learning algorithm approximates the Expectation-Maximization algorithm, and classifies the input data encoded into spatio-temporal spike patterns. Even in the supervised classification, the algorithm requires no external spikes indicating the desired spike timings unlike existing algorithms. Furthermore, because the algorithm is consistent with biological models and hypotheses found in existing biological studies, it could capture the mechanism underlying biological delay learning.

## 1. Introduction

As confirmed in biological studies, the precise timing of a neuronal spike plays a fundamental role in information processing in the central nervous system (Carr and Konishi, 1990; Middlebrooks et al., 1994; Seidl et al., 2010). In information coding called *temporal coding*, the timing of at least one generated spike represents an output. In contrast, in rate coding, spiking neural network (SNN) generates spikes repeatedly over a certain period and the number of generated spikes represents an output (Brader et al., 2007; Nessler et al., 2009; Beyeler et al., 2013; O'Connor et al., 2013; Diehl and Cook, 2015; Zambrano and Bohte, 2016). SNNs in rate coding are increasingly being investigated for efficient computational architectures in engineering applications. The SNN-based architectures consume less energy and require smaller hardware area than traditional artificial neural network architectures (Querlioz et al., 2013; Neftci et al., 2014; Cao et al., 2015). However, repeated spike generation increases the computational time (VanRullen and Thorpe, 2001). To improve the efficiency of SNN-based architectures, the SNN can be implemented in temporal coding rather than rate coding (Matsubara and Torikai, 2013, 2016).

**Figure 1:**
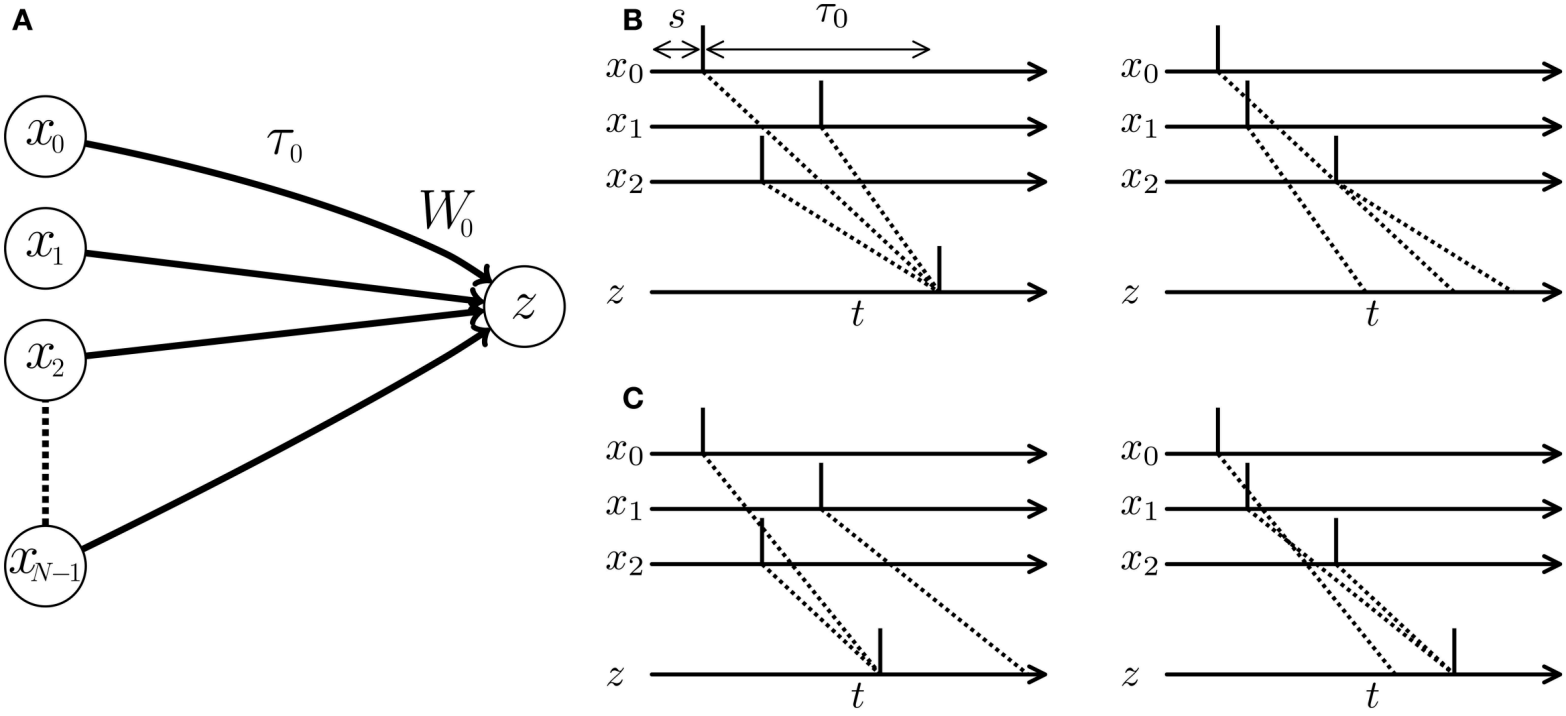
**(A)** Schematic of a spiking neural network (SNN) consisting of multiple pre-synaptic neurons *x*_*i*_ and a post-synaptic neuron *z. W*_0_ denotes the weight of the synapse projecting from the pre-synaptic neuron *x*_0_ to the post-synaptic neuron *z*, and τ_0_ denotes the conduction delay of the axon corresponding to the synapse. **(B,C)** Post-synaptic spikes in response to spatio-temporal spike patterns. Each vertical line denotes a spike elicited by a neuron. Dotted lines show the transmission paths of the pre-synaptic spikes to the post-synaptic neuron *z*. **(B)** When the conduction delays are fixed, the post-synaptic neuron responds to the spatio-temporal pattern of pre-synaptic spikes and elicits a spike (left panel), but is unresponsive to a second pattern (right panel). **(C)** When the conduction delays are plastic, the optimized SNN generates post-synaptic spikes at times depending on the given spatio-temporal patterns of the pre-synaptic spikes.

When multiple pre-synaptic spikes simultaneously arrive at a post-synaptic neuron, they evoke a large excitatory post-synaptic potential (EPSP). In response, the post-synaptic neuron elicits a spike and thereby delivers the signal to the latter part of the SNN with a high probability (see Figure 1). In other words, a neuron behaves as a coincidence detector. As the membrane potential of the post-synaptic neuron increases rapidly with increasing efficacy of the projecting synapse, synaptic modification influences the post-synaptic spike timing. Many algorithms adjust the timing of post-synaptic spikes in a supervised manner by potentiating synapses that potentially evoke EPSPs at the desired timing, while depressing other synapses. Examples are the ReSuMe algorithm (Ponulak, 2005; Sporea and Grüning, 2013; Matsubara and Torikai, 2016), the Tempotron algorithm (Gütig and Sompolinsky, 2006; Yu et al., 2014), and algorithms proposed in Pfister et al. (2006) and Paugam-Moisy et al. (2008). A pre-synaptic spike arrives at a post-synaptic neuron through the axon of the pre-synaptic neuron. The delay incurred by traveling through the axon is called the *axonal conduction delay* (Waxman and Swadlow, 1976). Thus, temporal coding must consider the pre-synaptic spike timing plus the conduction delay (see left panel of Figure 1B). Izhikevich et al. (2004) and Izhikevich (2006b) demonstrated that even when the conduction delays are constant, an SNN with synaptic modification called *spike timing-dependent plasticity* (STDP) (Markram et al., 1997; Bi and Poo, 1998) self-organizes its characteristic responses to spatio-temporal spike patterns. In Gerstner et al. (1996), multiple pre-synaptic neurons driven by a single signal source deliver the signal to a post-synaptic neuron after various delays. Synaptic modification maintains the synapses that simultaneously evoke an EPSP by potentiating them and prunes other synapses by depressing them. SpikeProp (Bohte et al., 2002) models a similar process in a supervised manner. However, under the assumption of multiple connections, the SNN requires numerous unused paths for future development. Alternatively, the SNN accepts limited spatio-temporal spike patterns depending on the initial network connections and conduction delays. Fixing the delay time limits the flexibility and efficiency of the SNN (see right panel of Figure 1B).

As mentioned above, information processing in a neural circuit requires the synchronous arrival of spikes elicited by multiple pre-synaptic neurons, and thus optimal conduction delay in the axons is critical. Axons are surrounded by *myelin*, which works as an electrical insulator and reduces the conduction delay (Rushton, 1951). Myelination and demyelination of axons appear to depend on neuronal activity (Fields, 2005; Bakkum et al., 2008; Jamann et al., 2017). Fields (2015) and Baraban et al. (2016) hypothesized that synaptic modification occurs only after the arrival timings of the pre-synaptic spikes have been adjusted by activity-dependent myelination.

The adaptation of the conduction delay is called *delay learning*. Supervised delay learning algorithms such as the DL-ReSuMe algorithm (Taherkhani et al., 2015) directly adjust the conduction delay to suit the given spatio-temporal patterns of the pre- and post-synaptic spikes. After the adjustment, the SNN generates post-synaptic spikes at the desired timings (see Figure 1C). These algorithms answer the purpose to reproduce the spatio-temporal patterns, but are not suited for classification of the spatio-temporal patterns because the desired timings are generally unknown and should be manually adjusted with great care in the classification tasks. Hence, this approach reduces the flexibility in the context of machine learning and poorly represents biological systems that self-adapt to changing environments. In unsupervised delay learning, the SNN instead self-organizes in response to a given spike pattern (Hüning et al., 1998; Eurich et al., 1999, 2000). However, none of the unsupervised delay learning algorithms tackled practical tasks such as classification and reproduction of given spike patterns (see Table 1 for comparison). Thus, a delay learning algorithm that classifies given spike patterns in both unsupervised and supervised manners is greatly desired.

**Table 1:** Comparison of SNNs in different studies.

**Studies**	**Weight**	**Delay**	**Coding**	**Learning manner**	**Classification**
Brader et al., 2007 and others^*^	Plastic	No	Rate	Unsupervised and/or supervised	Yes
Ponulak, 2005 and others ^**^	Plastic	No	Temporal	Supervised	Yes
Izhikevich et al., 2004; Izhikevich, 2006b	Plastic	Fixed	Temporal	Unsupervised	No
Gerstner et al., 1996	Plastic	Fixed/multiple	Temporal	Unsupervised	No
Bohte et al., 2002	Plastic	Fixed/multiple	Temporal	Supervised	Yes
Taherkhani et al., 2015	Plastic	Plastic	Temporal	Supervised	Yes
Hüning et al., 1998 and others ^***^	Plastic	Plastic	Temporal	Unsupervised	No
This study	Plastic	Plastic	Temporal	Unsupervised and supervised	Yes

* *Brader et al., 2007; Nessler et al., 2009; Beyeler et al., 2013; O'Connor et al., 2013; Querlioz et al., 2013; Neftci et al., 2014; Diehl and Cook, 2015; Zambrano and Bohte, 2016*.

** *Bohte et al., 2002; Ponulak, 2005; Gütig and Sompolinsky, 2006; Pfister et al., 2006; Paugam-Moisy et al., 2008; Matsubara and Torikai, 2016*.

*** *Hüning et al., 1998; Eurich et al., 1999, 2000*.

The present study proposes an unsupervised learning algorithm that adjusts the conduction delays and synaptic weights of an SNN. In a theoretical analysis, the proposed learning algorithm is confirmed to approximate the Expectation-Maximization (EM) algorithm. Optimization algorithms in probabilistic models including EM algorithm have been analyzed SNNs with no or fixed delay (e.g., Nessler et al., 2009; Kappel et al., 2014, 2015; Rezende and Gerstner, 2014). The proposed learning algorithm can be considered to extend the earlier methods to the conduction delay optimization. When evaluated on several practical classification tasks, the proposed algorithm successfully discriminated the given spatio-temporal spike patterns in an unsupervised manner. Furthermore, the algorithm was adaptable to supervised learning for improved classification accuracy. Remarkably, even in the supervised classification, the algorithm does give no external spikes indicating the timings at which the SNN should generate spikes. The adjustment of the synaptic weight in the proposed algorithm mimics that of STDP and occurs after the conduction delay was adjusted, supporting the hypothesis of Fields (2015) and Baraban et al. (2016). Therefore, the proposed learning algorithm presents as a good hypothetical model of biological delay learning, and might contribute to further investigations of SNNs in temporal coding. Preliminary and limited results of this study were presented in our conference paper (Matsubara, 2017).

## 2. Spike timing-dependent conduction delay learning

### 2.1. Spiking neural network (SNN)

An SNN consists of multiple pre-synaptic neurons *x*_0_, …, *x*_*N*−1_ connected to a post-synaptic neuron *z* (see Figure 1A). Let *T* be an experimental time period and δ be the time step. The discrete times are denoted by *t* = 0, δ, 2δ, …  (*t* < *T*). The binary variable *x*_*i,s*_ is set to 1 when a pre-synaptic neuron *x*_*i*_ generates a spike at time *s* and 0 otherwise. Owing to the discrete time, a spike has a width equal to the time step δ. Hence, each set ***x*** = {*x*_*is*_} represents a spatio-temporal spike pattern (see Figure 1B). The pre-synaptic neuron *x*_*i*_ is connected to the post-synaptic neuron *z* via a synapse with a synaptic weight *W*_*i*_ ≥ 0 and a conduction delay τ_*i*_ ≥ 0. A pre-synaptic spike *x*_*is*_ = 1 arrives at the post-synaptic neuron *z* after a conduction delay τ_*i*_. After τ_*i*_, the spike evokes an excitatory post-synaptic potential (EPSP) proportional to the synaptic weight *W*_*i*_. The time course of the EPSP is expressed by a temporal relation function *g*(Δ*t*) that depends on the temporal difference *s*−*t* and the conduction delay τ_*i*_ (namely, Δ*t* = *s* + τ_*i*_ − *t*). For multiple pre-synaptic spikes, the EPSP is the linear sum of the EPSPs evoked by the pre-synaptic spikes. Then, the post-synaptic membrane potential *v*_*t*_ at time *t* is then given by

(1) $$\begin{array}{l} v_{t}=\underset{is}{\sum }x_{is}W_{i}g\left(s+\tau_{i}-t\right). \end{array}$$

The binary variable *z*_*t*_ is set to 1 when the post-synaptic neuron *z* generates a spike at time *t* and 0 otherwise. For the parameters **τ** = {τ_*i*_} and ***W*** = {*W*_*i*_} and a given spatio-temporal spike pattern ***x*** = {*x*_*is*_}, the post-synaptic neuron *z* is assumed to generate a single spike within the experimental time period 0 ≤ *t* < *T*, i.e., $\underset{t}{\sum }z_{t}=1$. Such a constraint is important and is commonly applied in studies of temporal coding (e.g., Bohte et al., 2002) where the output of the SNN in temporal coding is defined as the timing of the post-synaptic spike. The present study initially follows the previous studies, but later proposed an alternative model without the constraint (see section 2.5). This study also assumes that for a spatio-temporal spike pattern ***x*** = {*x*_*is*_}, the probability of generating a post-synaptic spike *z*_*t*_ = 1 at time *t* is exponentially proportional to the EPSP normalized by a constant $Z=\underset{t^{\prime }}{\sum }\exp\left(v_{t^{\prime }}\right)$ as follows:

(2) $$\begin{array}{l} p(z_{t}=1|x)=\frac{\exp(v_{t})}{\sum_{t^{\prime }}\exp(v_{t})}\\ \;=\frac{1}{Z}\exp(v_{t})\\ \;\propto \exp\left(\sum_{is}x_{is}W_{i}g(s+\tau_{i}-t)\right). \end{array}$$

Note that *p*(*z*_*t*_ = 1|***x***) > 0 at any time *t*. In other words, the post-synaptic neuron *z* can elicit a spike even before the first pre-synaptic spike arrives. In biological neural networks, such spikes are induced by physiological noise. Note also that the normalizing constant *Z* includes a term $v_{t^{\prime }}$ denoting the membrane potential at a future time *t*′ > *t*. Therefore, sampling the post-synaptic spike *z*_*t*_ = 1 requires anti-causal information. The alternative model proposed in section 2.5 overcomes this limitation.

### 2.2. Multinoulli-Bernoulli mixture model

This subsection introduces the mixture probabilistic model. Let ***x*** be a set of visible binary random variables {*x*_*is*_}, each following a Bernoulli distribution, and ***z*** be a set of latent binary random variables {*z*_*t*_} following a Multinoulli distribution, i.e., $\underset{t}{\sum }z_{t}=1$. Also let **ω** = {ω_*t*_} denote the mixture weights, and **π** = {π_*ist*_} denote the posterior probability of *x*_*is*_ = 1 given *z*_*t*_ = 1. The generative Multinoulli-Bernoulli mixture model *p*(***z***, ***x***; **ω**, **π**) is then expressed as

$$\begin{array}{l} \;p(z,x;\omega ,\pi )=\prod_{t}{\left[\omega_{t}\prod_{is}{\left(\pi_{ist}\right)}^{x_{is}}{\left(1-\pi_{ist}\right)}^{1-x_{is}}\right]}^{z_{t}},\\ \text{log }p(z,x;\omega ,\pi )=\sum_{t}\;\;z_{t}[\text{log }\omega_{t}+\sum_{is}x_{is}\text{log }\frac{\pi_{ist}}{1-\pi_{ist}}\\ \;+\sum_{is}\text{log }(1-\pi_{ist})]. \end{array}$$

Here, the posterior probability π_*ist*_ is substituted by sigm(*V*_*ist*_) using the sigmoid function sigm(*u*) = (1+exp(−*u*))^−1^. In addition, *V*_*ist*_ is modeled by the function *W*_*i*_*g*(*s* + τ_*i*_ − *t*)−*v* where *v* is a bias parameter and the mixture weight *w*_*t*_ is assumed to have a constant value *e*^*b*^. The log-probability is then rewritten as

(3) $$\begin{array}{l} \text{log }p(z,x;\omega ,\pi )=\sum_{t}\;z_{t}\left[\text{log }\omega_{t}+\sum_{is}x_{is}V_{ist}-\sum_{is}\text{log }(1+e^{V_{ist}})\right]\\ \;=\sum_{t}\;z_{t}[b+\sum_{is}x_{is}(W_{i}g(s+\tau_{i}-t)-v)\\ \;-\sum_{is}\text{log }\left(1+e^{\left(W_{i}g(s+\tau_{i}-t)-v\right)}\right)]\\ \;=\text{log }p(z,x;W,\tau ). \end{array}$$

When the experimental time period *T* is sufficiently long and the time *t* is sufficiently distant from the temporal boundaries (0 and *T*), $\hat{b}$ is independent of the post-synaptic spike timing *t*; that is, $\hat{b}=b-\underset{is}{\sum }\text{log }\left(1+e^{W_{i}g\left(s+\tau_{i}-t\right)-v}\right)$. The posterior probability of *z*_*t*_ = 1 given ***x*** = {*x*_*is*_} is then equivalent to Equation (2);

$$\begin{array}{l} p\left(z_{t}=1|x;W,\tau \right)=\frac{p\left(z_{t}=1,x\right)}{\sum_{t^{\prime }}p\left(z_{t^{\prime }}=1,x\right)}\\ \propto \exp\left(\hat{b}+\underset{is}{\sum }x_{is}\left(W_{i}g\left(s+\tau_{i}-t\right)-v\right)\right)\\ \propto \exp\left(\underset{is}{\sum }x_{is}W_{i}g\left(s+\tau_{i}-t\right)\right). \end{array}$$

### 2.3. Learning algorithm based on the EM algorithm

The SNN model Equation (2) can be optimized through optimization of the Multinoulli-Bernoulli mixture model Equation (4) under the certain constraints. Let θ be the parameter set and ***X*** be a dataset of spatio-temporal spike patterns ***x***. In general, training a generative model involves maximizing the model evidence 𝔼_***x***~***X***_[log *p*(***x***; **θ**)] (Murphy, 2012). For an arbitrary distribution *q*(***z***), the evidence is expressed as

$$\begin{array}{l} {𝔼}_{x\sim X}\left[\text{log }p\left(x;\theta \right)\right]={𝔼}_{x\sim X}{𝔼}_{z\sim q\left(z\right)}\left[\text{log }\frac{p\left(z,x;\theta \right)q\left(z\right)}{p\left(z|x;\theta \right)q\left(z\right)}\right]\\ ={𝔼}_{x\sim X}{𝔼}_{z\sim q\left(z\right)}\left[\text{log }p\left(z,x;\theta \right)-\text{log }q\left(z\right)\right]\\ +\;{𝔼}_{x\sim X}\left[D_{KL}\left(q\left(z\right)||p\left(z|x;\theta \right)\right)\right]\\ =L\left(\theta \right)+{𝔼}_{x\sim X}\left[D_{KL}\left(q\left(z\right)||p\left(z|x;\theta \right)\right)\right], \end{array}$$

where the evidence lower bound L(θ) equals 𝔼_***x***~***X***_𝔼_***z***~*q*(***z***)_[log *p*(***z***, ***x***; **θ**)−log *q*(***z***)] and *D*_*KL*_(·) is the Kullback-Leibler divergence. The model evidence 𝔼_***x***~***X***_[log *p*(***x***; **θ**)] can be maximized by the Expectation-Maximization (EM) algorithm. In the E-step, *q*(***z***) is substituted by the posterior probability *p*(***z***|***x***; **θ**^*old*^) of the mixture model with the currently estimated parameter set **θ**^*old*^. The M-step updates the estimated parameter set **θ** to maximize the evidence lower bound L(θ). The present study employs a stochastic version of the EM algorithm (Sato, 1999; Nessler et al., 2009). Given a spatio-temporal spike pattern $\hat{x}$, the stochastic EM algorithm first generates a post-synaptic spike $\hat{z}$ by Equation (2). This spike generation corresponds to the E-step of the EM algorithm. Next, the parameters θ = {***W***, **τ**} are updated to maximize the evidence lower bound L(θ) using a gradient ascent algorithm. This parameter update corresponds to the M-step of the EM algorithm. From an SNN perspective, the parameter update corresponds to synaptic plasticity with delay learning. Note that, unlike the original EM algorithm, the M-step of the stochastic EM algorithm is not guaranteed to maximize the model evidence 𝔼_***x***~***X***_[log *p*(***x***; **θ**)] but at least maximizes the evidence lower bound L(θ).

Based on Equation (4), the gradients of the evidence lower bound L(θ) w.r.t. the conduction delay τ_*i*_ and synaptic weight *W*_*i*_ are respectively given by;

(4) $$\begin{array}{l} \frac{\partial }{\partial \tau_{i}}L(\theta )=\sum_{t}z_{t}\sum_{s}(x_{is}-\text{sigm}\left(W_{i}g(s+\tau_{i}-t)-v)\right)\\ \;\times \;g(s+\tau_{i}-t)W_{i}\frac{-(s+\tau_{i}-t-\mu )}{\sigma^{2}}\\ \;=-\sum_{t}z_{t}\sum_{s}x_{is}\cdot g(s+\tau_{i}-t)W_{i}\frac{s+\tau_{i}-t-\mu }{\sigma^{2}}\\ \;=-\sum_{t}z_{t}\sum_{s}x_{is}\cdot g(\Delta t)W_{i}\frac{\Delta t-\mu }{\sigma^{2}}, \end{array}$$

(5) $$\begin{array}{l} \frac{\partial }{\partial W_{i}}L(\theta )\\ \;=\sum_{t,s}z_{t}(x_{is}-\text{sigm}(W_{i}g(s+\tau_{i}-t)-v))g(s+\tau_{i}-t)\\ \;=\left[\sum_{t,s}z_{t}x_{is}g(s+\tau_{i}-t)\right]-\left[\sum_{\Delta t^{\prime }}\;\text{sigm}(W_{i}g(\Delta t^{\prime })-v)g(\Delta t^{\prime })\right]\\ \;=\left[\sum_{t,s}z_{t}x_{is}g(\Delta t)\right]-\left[\sum_{\Delta t^{\prime }}\text{sigm}(W_{i}g(\Delta t^{\prime })-v)g(\Delta t^{\prime })\right]. \end{array}$$

As Equation (4) and the first term in Equation (5) are independent of the time step δ, the second term in Equation (5) (which does depend on δ) is multiplied by δ: This normalization makes Equation (5) robust to the size of the time step δ. The parameters θ are then updated as follows:

$$\begin{array}{l} \theta \leftarrow \theta +\eta \frac{\partial }{\partial \theta }L\left(\theta \right){|}_{z=\hat{z},x=\hat{x}}, \end{array}$$

where η is a learning rate. The conditions are similar in a supervised manner, except that the post-synaptic spike $\hat{z}$ is sourced externally.

### 2.4. Classification

A spatio-temporal spike pattern ***x*** is associated with one of the timings *t* = 0δ, 1δ, …  depending on the timing *t* of the generated post-synaptic spike *z*_*t*_ = 1. However, a given dataset ***X*** of spatio-temporal spike patterns ***x*** should be classifiable into smaller groups. In this study, the decision boundaries between *N* groups are defined as the *n*-th *N*-quantiles (*n* = 1, 2, …, *N* − 1) of the generated post-synaptic spike timings *t* for which *z*_*t*_ = 1. For example, suppose that a dataset ***X*** contains 100 spatio-temporal spike patterns ***x*** falling into estimated two groups. The spatio-temporal spike patterns ***x*** are then sorted by the timings *t* of the post-synaptic spikes *z*_*t*_ = 1. After sorting, patterns 1–50 are classified into group 1 and the remainder are classified into group 2.

### 2.5. Alternative spiking neural network

In the SNN introduced above, the Multinoulli distribution *p*(***z***|***x***; **τ**, ***W***) must be sampled over time *t* to generate a post-synaptic spike *z*_*t*_ = 1. In other words, whether a post-synaptic spike *z*_*t*_ = 1 is generated at time *t* anti-causally depends on the EPSP $v_{t^{\prime }}$ and post-synaptic spike $z_{t^{\prime }}$ at a future time *t*′ > *t*. To remove this unrealistic assumption, this subsection slightly modifies the SNN formulation. The new formulation replaces the Multinoulli distribution with multiple independent Bernoulli distributions and introduces the following Bernoulli–Bernoulli mixture model with posterior probability **ρ** = {ρ_*ist*_} of *x*_*is*_ = 1 given *z*_*t*_ = 0;

$$\begin{array}{l} p\left(z_{t},x;\omega ,\pi ,\rho \right)={\left[\omega_{t}\underset{is}{\prod }{\left(\pi_{ist}\right)}^{x_{is}}{\left(1-\pi_{ist}\right)}^{1-x_{is}}\right]}^{z_{t}}\\ \times {\left[\left(1-\omega_{t}\right)\underset{is}{\prod }{\left(\rho_{ist}\right)}^{x_{is}}{\left(1-\rho_{ist}\right)}^{1-x_{is}}\right]}^{1-z_{t}}. \end{array}$$

In this case, given a spatio-temporal spike pattern ***x***, the posterior probabilities *p*(*z*_*t*_|***x***; **ω**, **π**, **ρ**) of the post-synaptic spikes *z*_*t*_ = 1 at times *t* are completely independent. As described above, the prior probability ω_*t*_ of *z*_*t*_ = 1 is replaced with a time-independent term *e*^*b*^ and the posterior probability π_*ist*_ of *x*_*is*_ = 1 given *z*_*t*_ = 1 is modeled as π_*ist*_ = sigm(*V*_*ist*_) = sigm(*W*_*i*_*g*(*s* + τ_*i*_ − *t*)−*v*). In addition, the posterior probability ρ_*ist*_ is substituted by a constant sigm(*v*). The log-probability function becomes

$$\begin{array}{l} \text{log }p(z_{t},x;\omega ,\pi ,\rho )\\ \;=z_{t}\left[\text{log }\omega_{t}+\sum_{is}x_{is}\text{log }\frac{\pi_{ist}}{1-\pi_{ist}}+\sum_{is}\text{log }(1-\pi_{ist})\right]\\ \;+(1-z_{t})[\text{log }(1-\omega_{t})+\sum_{is}x_{is}\text{log }\frac{\rho_{ist}}{1-\rho_{ist}}\\ \;+\sum_{is}\text{log }(1-\rho_{ist})]\\ \;=z_{t}\left[\text{log }\frac{\omega_{t}}{1-\omega_{t}}+\sum_{is}x_{is}\left(V_{ist}+v\right)-\sum_{is}\text{log }\frac{1+e^{V_{ist}}}{1+e^{-v}}\right]\\ \;+\left[\text{log }(1-\omega_{t})-v\sum_{is}x_{is}+\sum_{is}\text{log }(1+e^{-v})\right]\\ \;=z_{t}[\hat{b}+\sum_{is}x_{is}W_{i}g(s+\tau_{i}-t)\\ \;-\sum_{is}\text{log }\left(1+e^{W_{i}g(s+\tau_{i}-t)-v}\right)]+\hat{c}\\ \;=\text{log }p(z_{t},x;W,\tau ), \end{array}$$

where $\hat{b}=\text{log }\frac{\omega_{t}}{1-\omega_{t}}+\underset{is}{\sum }\text{log }\left(1+e^{-v}\right)$ and $ĉ=\text{log }\left(1-\omega_{t}\right)-v\underset{is}{\sum }x_{is}+\underset{is}{\sum }\text{log }\left(1+e^{-v}\right)$. The prior probability ω_*t*_ is replaced by an independent variable $\hat{b}$ and a variable ĉ that depends on $\hat{b}$ and *v*.

The posterior probability of *z*_*t*_ = 1 given ***x*** is expressed as

(6) $$\begin{array}{l} p(z_{t}=1|x;W,\tau )=\text{sigm}(\text{log }p(z_{t}=1,x;W,\tau )\\ \;-\text{log }p(z_{t}=0,x;W,\tau ))\\ \;=\text{sigm}\left(\hat{b}+\sum_{is}x_{is}\left(W_{i}g(s+\tau_{i}-t\right)\right)\\ \;-\sum_{is}\text{log }\left(1+e^{W_{i}g(s+\tau_{i}-t)-v})\right)\\ \;=\text{sigm}\left(\sum_{is}x_{is}W_{i}g(s+\tau_{i}-t)+\tilde{b}\right), \end{array}$$

where $\tilde{b}=\hat{b}-\underset{is}{\sum }\text{log }\left(1+e^{W_{i}g\left(s+\tau_{i}-t\right)-v}\right)$. The variable $\hat{b}$ is replaced by a variable $\tilde{b}$, which is treated as an independent parameter hereafter. The independent parameter *b* in the Bernoulli–Bernoulli mixture model is additional to the parameters in the Multinoulli-Bernoulli mixture model.

The evidence lower bound L(θ) is given by 𝔼_***x***~***X***_𝔼_*z*_*t*_~*q*(*z*_*t*_)_[log *p*(*z*_*t*_, ***x***; **θ**)−log *q*(*z*_*t*_)]]. The gradients of L(θ) w.r.t. the parameters τ_*i*_ and *W*_*i*_ are, respectively given by

(7) $$\begin{array}{l} \frac{\partial }{\partial \tau_{i}}L(\theta )=z_{t}\sum_{s}(x_{is}-\text{sigm}(W_{i}g(s+\tau_{i}-t)-v))\\ \;\times \;g(s+\tau_{i}-t)W_{i}\frac{-(s+\tau_{i}-t-\mu )}{\sigma^{2}}\\ \;=-z_{t}\sum_{s}x_{is}\cdot g(s+\tau_{i}-t)W_{i}\frac{s+\tau_{i}-t-\mu }{\sigma^{2}}\\ \;=-z_{t}\sum_{s}x_{is}\cdot g(\Delta t)W_{i}\frac{\Delta t-\mu }{\sigma^{2}}, \end{array}$$

(8) $$\begin{array}{l} \frac{\partial }{\partial W_{i}}L(\theta )=\sum_{s}z_{t}(x_{is}-\text{sigm}(W_{i}g(s+\tau_{i}-t)-v))g(s+\tau_{i}-t)\\ \;=\left[\sum_{s}z_{t}x_{is}g(s+\tau_{i}-t)\right]-\left[\sum_{\Delta t^{\prime }}\;\text{sigm}(W_{i}g(\Delta t^{\prime })-v)g(\Delta t^{\prime })\right]\\ \;=\left[\sum_{s}z_{t}x_{is}g(\Delta t)\right]-\left[\sum_{\Delta t^{\prime }}\text{sigm}(W_{i}g(\Delta t^{\prime })-v)g(\Delta t^{\prime })\right]. \end{array}$$

Equations (7) and (8) are equivalent to Equations (4) and (5) respectively, but are summed over time *t* (they sum the post-synaptic spikes *z*_*t*_ = 1). Therefore, given a spatio-temporal spike pattern ***x*** and a single post-synaptic spike *z*_*t*_ = 1, the parameters are updated as in the Multinoulli-Bernoulli mixture model.

Unlike the SNN based on the Multinoulli-Bernoulli mixture model, the above SNN is unconstrained by the number of post-synaptic spikes (i.e., $\underset{t}{\sum }z_{t}\in \mathbb{Z}$). To prevent an excessive number or the complete absence of post-synaptic spikes, this subsection introduces *homeostatic plasticity* (Turrigiano et al., 1999; Turrigiano and Nelson, 2000), a biological mechanism that presumably maintains the excitability of a neuron within a regular range (Van Rossum et al., 2000; Abraham, 2008; Watt and Desai, 2010; Matsubara and Uehara, 2016). The additional parameter $\tilde{b}$ in Equation (6) embodies the intrinsic excitability of the Bernoulli neuron *z*. The intrinsic excitability $\tilde{b}$ is adjusted after every receipt of the spatio-temporal spike pattern ***x***. When post-synaptic neuron *z* generates at least one spike (i.e., $\underset{t}{\sum }z_{t}>0$), its intrinsic excitability $\tilde{b}$ reduces by ${\tilde{b}}_{-}$; conversely if no spikes are generated (i.e., $\underset{t}{\sum }z_{t}=0$), its intrinsic excitability $\tilde{b}$ increases by ${\tilde{b}}_{+}$. Algorithmically, this conditional expression is given by

$$\begin{array}{l} \tilde{b}\leftarrow \{\begin{array}{ll} \tilde{b}-{\tilde{b}}_{-} & \text{if}\sum_{t}z_{t}>0\\ \tilde{b}+{\tilde{b}}_{+} & \text{otherwise}. \end{array} \end{array}$$

The spatio-temporal spike patterns ***x*** are classified as described in section 2.4, with an important difference; each post-synaptic spike *z*_*t*_ = 1 is weighted by the inverse number of generated post-synaptic spikes, i.e., $1/\underset{t}{\sum }z_{t}$. In other words, a spatio-temporal spike pattern ***x*** is associated with a group by majority voting of multiple post-synaptic spikes *z*_*t*_ = 1.

## 3. Experiments and results

### 3.1. Windows of plasticity

In this study, the temporal relation function *g*(Δ*t*) was expressed as the following Gaussian function:

(9) $$\begin{array}{l} g\left(\Delta t\right)=\frac{1}{\sqrt{2\pi }\sigma }\exp\left(-\frac{{\left(\Delta t-\mu \right)}^{2}}{2\sigma^{2}}\right). \end{array}$$

**Figure 2:**
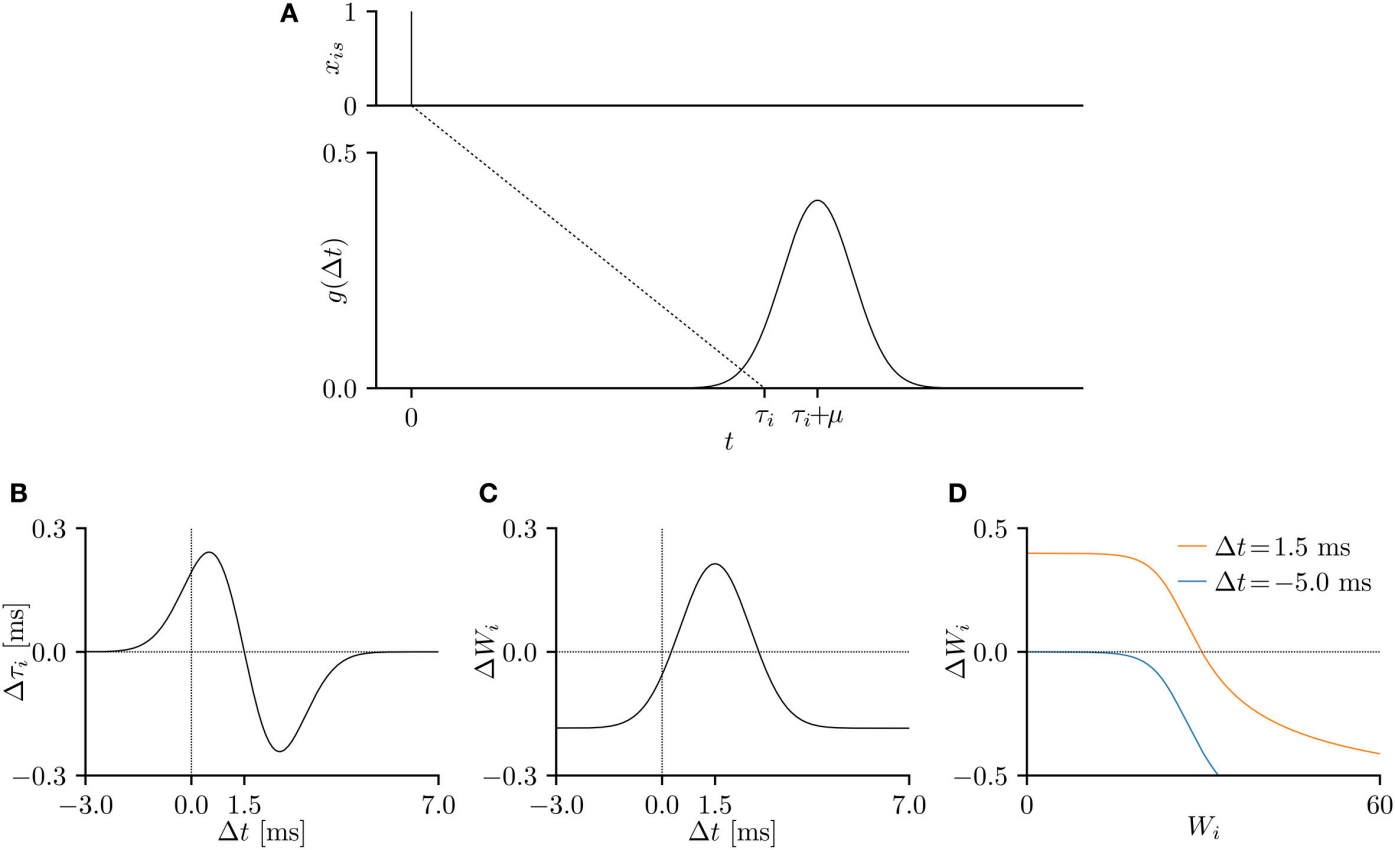
**(A)** A temporal relation function *g*(Δ*t*), representing an excitatory post-synaptic potential (EPSP) or current (EPSC), when the pre-synaptic spike is elicited at *s* = 0 ms. The parameters were set to μ = 1.5 ms and σ = 1.0 ms and the conduction delay τ_*i*_ was set to τ = 10 ms. **(B–D)** The gradients of the lower bound L(θ) of the model evidence 𝔼_***x***~***X***_[log *p*(***x***; **θ**)] with respect to the parameters *W*_*i*_ and τ_*i*_. The time step δ was 0.05 ms, the experimental time period *T* was 50 ms, and the remaining parameters were set to *v* = 10, and η = 0.001. The gradient shapes depend on the shape of the temporal relation function *g*(Δ*t*). **(B)** Temporal window of the delay learning, showing the relationship between the temporal difference Δ*t* and the change Δτ_*i*_ in conduction delay τ_*i*_, where Δ*t* = *s* + τ_*i*_ − *t*. **(C)** Temporal window of the synaptic plasticity, showing the relationship between the temporal difference Δ*t* and the change Δ*W*_*i*_ in synaptic weight *W*_*i*_. **(D)** The change Δ*W*_*i*_ in synaptic weight *W*_*i*_ versus the current synaptic weight *W*_*i*_ for Δ*t* = 1.5 ms (orange) and Δ*t* = -5.0 ms (blue).

Here, the parameters μ and σ denote the mean and standard deviation of the distribution, respectively. Figure 2A plots the temporal relation function *g*(Δ*t*) when μ = 1.5 ms and σ = 1 ms. The EPSP is commonly modeled by the double-exponential function (e.g., Shouval et al., 2010), which has zero gradient for all Δ*t* < 0. As the proposed SNN is trained according to the gradient of the temporal relation function *g*(Δ*t*), the double-exponential function is unsuitable for the purpose. In this study, the causality was preserved by clamping the temporal relation function *g*(*s* + τ_*i*_ − *t*) to 0 for all *s* − *t* < 0.

The experimental time period *T* was 50 ms, the time step δ was set to 0.05 ms. The remaining parameters were set to μ = 1.5 ms, σ = 1.0 ms, *v* = 10, and η = 0.001, unless otherwise stated. The gradients of the evidence lower bound L(θ) w.r.t. the conduction delay τ_*i*_ and synaptic weight *W*_*i*_ were considered to be changes by the delay learning and synaptic plasticity, respectively. When plotted against the temporal difference Δ*t* = *s*+τ_*i*_−*t*, the gradients represent the temporal windows of the delay learning and synaptic plasticity. As shown in Figure 2B, the conduction delay τ_*i*_ increases (decreases) when the temporal difference Δ*t* is smaller (larger) than 1.5 ms. The EPSP peaks at 1.5 ms, indicating that a post-synaptic spike *x*_*t*_ = 1 attracts the nearby pre-synaptic spikes *x*_*is*_ = 1. The temporal window of the synaptic weight *W*_*i*_ is similar to that of the STDP (Figure 2C). Specifically, the synapse is potentiated (depressed) when the temporal difference Δ*t* is positive (negative), but is always depressed when the temporal difference Δ*t* is large and positive, as noted in previous electrophysiological studies (Markram et al., 1997; Bi and Poo, 1998) and theoretical studies (Shouval et al., 2002; Wittenberg and Wang, 2006; Shouval et al., 2010). Figure 2D plots the amount of synaptic modification Δ*W*_*i*_ versus the current synaptic weight *W*_*i*_. The decreasing trend is again consistent with previous electrophysiological (Bi and Poo, 1998) and theoretical (Van Rossum et al., 2000; Gütig et al., 2003; Shouval et al., 2010; Matsubara and Uehara, 2016) studies.

### 3.2. Results for toy spike patterns

**Figure 3:**
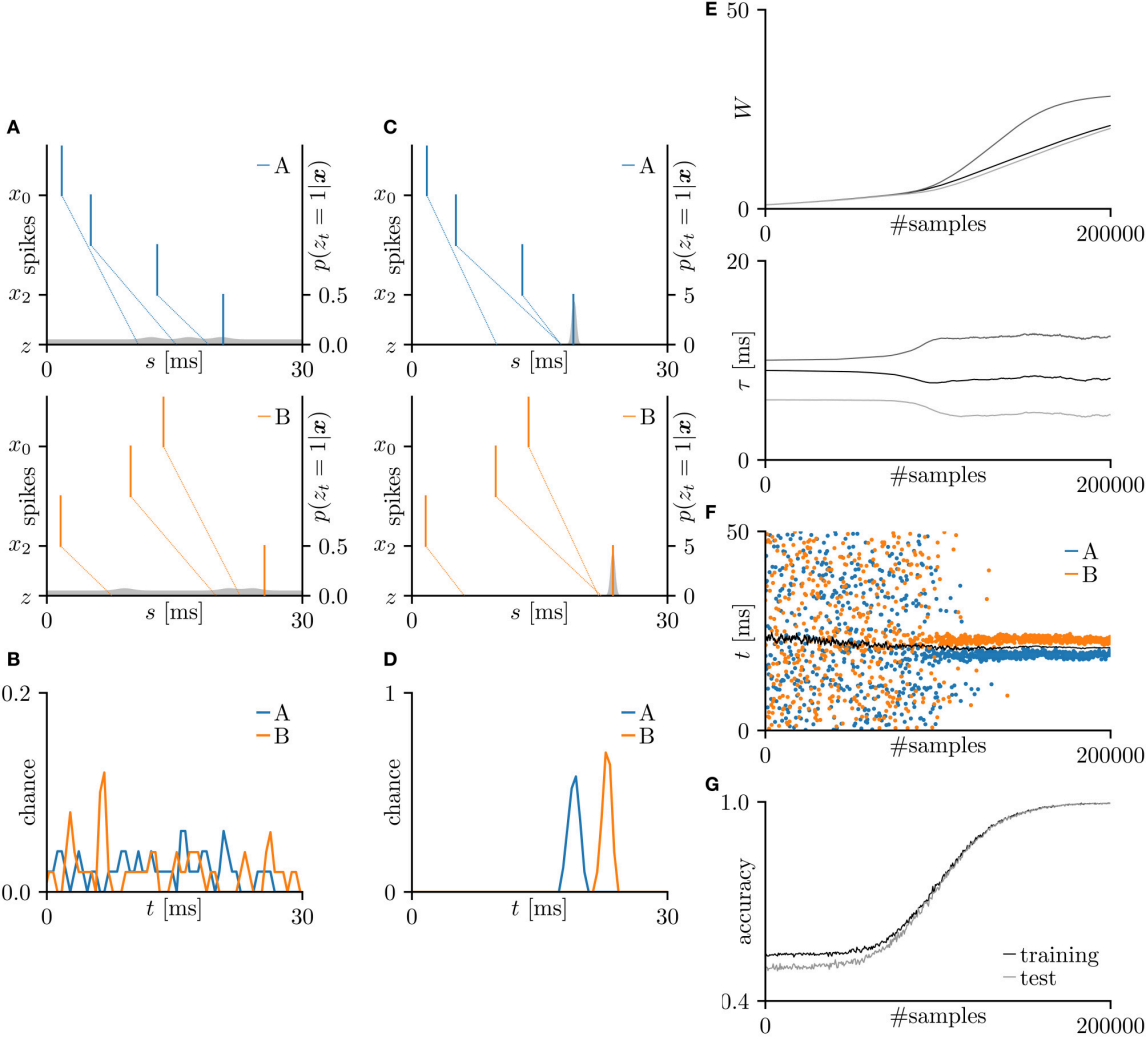
Results of SNN based on the Multinoulli-Bernoulli mixture model, evaluated on toy spike patterns. **(A)** Representative spatio-temporal spike patterns A (top panel) and B (bottom panel), and the post-synaptic spikes *z*_*t*_ = 1 before the learning procedure. Vertical lines represent pre-synaptic spikes *x*_*is*_ = 1 and post-synaptic spikes *z*_*t*_ = 1 (the left vertical axes). Dotted lines denote transmission of the pre-synaptic spikes *x*_*is*_ = 1 to the post-synaptic neuron *z* with conduction delays τ_*i*_. Gray shaded areas denote the probability of eliciting a post-synaptic spikes *z*_*t*_ = 1 per 1 ms (the right vertical axes). **(B)** Test distributions of the post-synaptic spike timings *z*_*t*_ = 1 before learning. **(C)** Post-synaptic spike timings *z*_*t*_ = 1 after learning, given the spatio-temporal spike patterns ***x*** in **(A)**. **(D)** Test distributions of the post-synaptic spike timing *z*_*t*_ = 1 after learning. **(E)** Trajectories of the synaptic weights *W*_*i*_ and conduction delays τ_*i*_ during the learning procedure. The darkest lines denote the values of the parameters *W*_0_ and τ_0_, and the lighter lines denote *W*_*i*_ and τ_*i*_ for *i* = 1, 2, … . **(F)** Random samples of the post-synaptic spike timings *z*_*t*_ = 1. The black solid line marks the decision boundary between spike patterns A and B. **(G)** Classification accuracy in the training and test datasets, each averaged over 100 trials.

First, the SNN and the proposed learning algorithm based on the Multinoulli-Bernoulli mixture model were evaluated on toy spike patterns generated by three pre-synaptic neurons. Figure 3A shows typical spatio-temporal spike patterns ***x***. The pre-synaptic neurons *x*_0_, *x*_1_, and *x*_2_ elicited spikes at 1+ξ_0_, 5+ξ_1_, and 13+ξ_2_ ms respectively in spike pattern A, and at 13+ξ_0_, 9+ξ_1_, and 1+ξ_2_ ms, respectively in spike pattern B. Here, ξ_0_, ξ_1_, and ξ_2_ are the noise terms following a uniform distribution U(-1,1). Fifty samples from each of spike patterns A and B were extracted as the training dataset; other fifty samples were reserved as the test dataset. All synaptic weights *W*_*i*_ were uniformly initialized to 1, and the conduction delays τ_*i*_ (ms) were sampled from the uniform distribution U(5,15). The post-synaptic neuron *z* was repeatedly fed with the spatio-temporal spike patterns ***x***, and generated spikes *z*_*t*_ = 1. The gray shaded areas in Figure 3A depicts the posterior probability *p*(*z*_*t*_ = 1|***x***; ***W***, **τ**) of spiking each millisecond. Figure 3B shows the probability distribution of post-synaptic spike timings *z*_*t*_ = 1 for the test dataset before learning. The unlearned model cannot discriminate the spike patterns A and B using the post-synaptic spike timings *z*_*t*_ = 1. Note that the proposed SNN is intrinsically probabilistic, meaning that a post-synaptic neuron can elicit a spike at an arbitrary time *t*. During the learning procedure, the synaptic weights *W*_*i*_ and the conduction delays τ_*i*_ were gradually changed by the proposed learning algorithm (Figure 3E). The conduction delays τ_*i*_ (ms) were clamped to the range 0–20 ms but never reached the limits. Figure 3F shows random samples of the post-synaptic spike timings *z*_*t*_ = 1, separated into spike patterns A and B by the decision boundary (the solid black line). After sufficiently many samples, the spike patterns converged into limited temporal ranges and the two groups were clearly demarcated. The post-synaptic spike timings *z*_*t*_ = 1 and their distributions after the learning procedure are exemplified in Figures 3C,D, respectively. In this trial, the classification accuracy of both the training and test datasets converged to 100 %. The average classification accuracy over 100 trials was 99.6 ± 2.6% for the training dataset and 99.6 ± 2.5% for the test dataset (Figure 3G). The results are summarized in Table 2.

**Table 2:** Classification accuracy of the proposed SNN based on the Multinoulli-Bernoulli mixture model.

**Dataset**	**Unsupervised**		**Supervised**	
	**Training**	**Test**	**Training**	**Test**
Toy spike patterns	99.6 ± 2.6	99.6 ± 2.5	100.0 ± 0.0	99.8 ± 1.2
Iris flower dataset	89.5 ± 5.3	89.5 ± 7.5	93.9 ± 5.3	89.4 ± 9.1
MNIST dataset	88.7 ± 3.4	88.7 ± 3.7	90.2 ± 4.0	90.1 ± 4.1

*All the classification accuracies are expressed as ave. ± std. (%)*.

### 3.3. Results for the iris flower dataset

**Figure 4:**
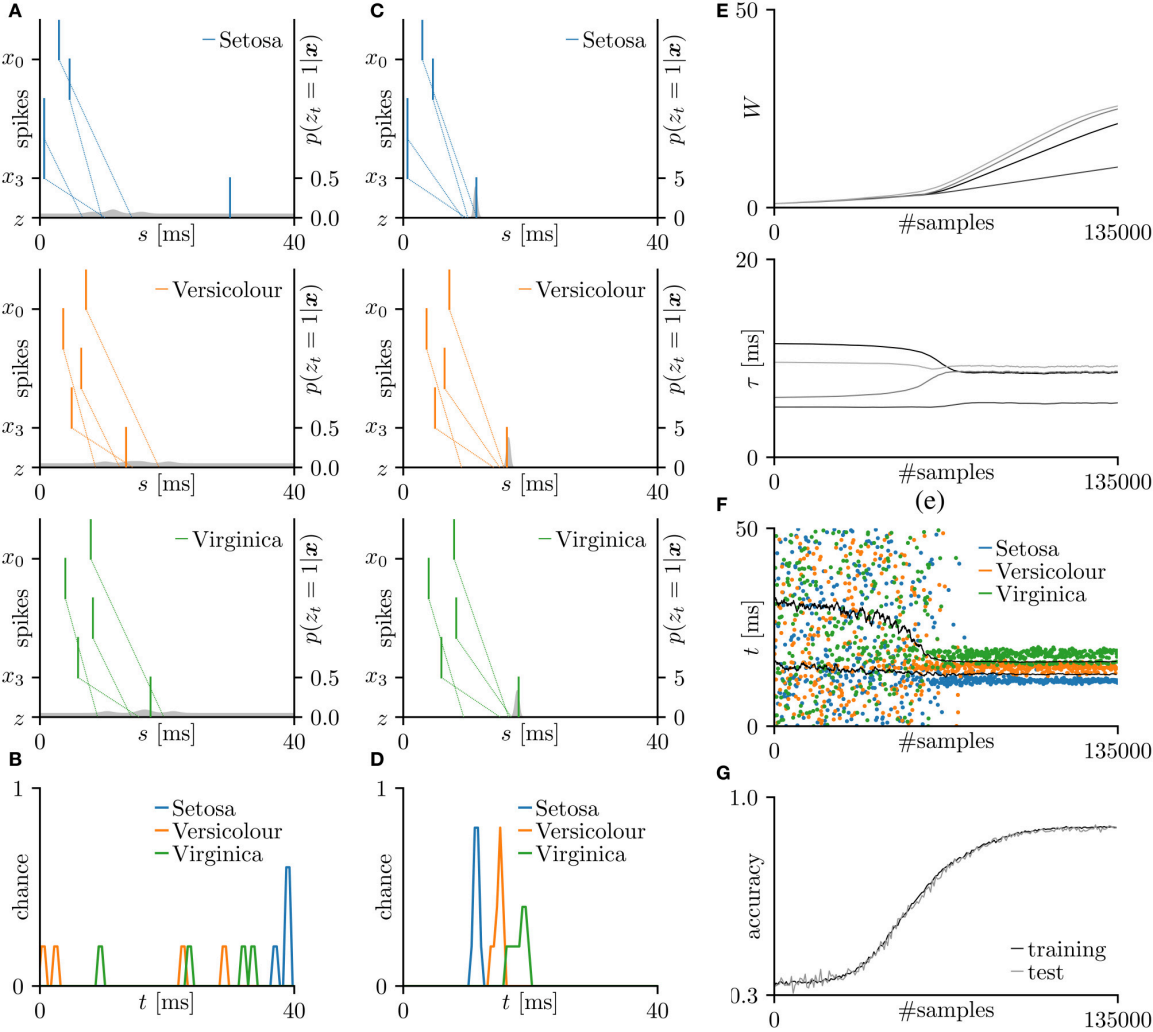
Results of SNN based on the Multinoulli-Bernoulli mixture model, evaluated on the iris flower dataset (Fisher, 1936). **(A)** Representative spatio-temporal spike patterns of Setosa (top panel), Versicolor (middle panel), and Virginica (bottom panel), and the post-synaptic spikes *z*_*t*_ = 1 before learning. Vertical lines represent pre-synaptic spikes *x*_*is*_ = 1 and post-synaptic spikes *z*_*t*_ = 1 (the left vertical axes). Dotted lines denote transmission of the pre-synaptic spikes *x*_*is*_ = 1 to the post-synaptic neuron *z* with conduction delays τ_*i*_. Gray shaded areas denote the probability of eliciting a post-synaptic spikes *z*_*t*_ = 1 per 1 ms (the right vertical axes). **(B)** Test distributions of the post-synaptic spike timings *z*_*t*_ = 1 before learning. **(C)** Post-synaptic spike timings *z*_*t*_ = 1 after learning, given the spatio-temporal spike patterns ***x*** in **(A)**. **(D)** Test distributions of the post-synaptic spike timing *z*_*t*_ = 1 after learning. **(E)** Trajectories of the synaptic weights *W*_*i*_ and conduction delays τ_*i*_ during the learning procedure. The darkest lines denote the values of the parameters *W*_0_ and τ_0_, and the lighter lines denote *W*_*i*_ and τ_*i*_ for *i* = 1, 2, … . **(F)** Random samples of the post-synaptic spike timings *z*_*t*_ = 1. The black solid lines mark the decision boundaries between the three species. **(G)** Classification accuracy in the training and test datasets, each averaged over 100 trials.

Next, the proposed learning algorithm was evaluated on the iris flower dataset (Fisher, 1936), which consists of the data of three species (Setosa, Versicolor, and Virginica). The dataset of each species comprises 50 data points, and each data point consists of four features (the lengths and widths of the sepals and petals). Accordingly, the number *N* of pre-synaptic neurons was set to 4. The spike timing of each feature was normalized to the range 0–10 ms. As the sepal length *k*_0_ ranges from 4.3 to 7.9 cm, the pre-synaptic neuron *x*_0_ generated a single spike at $s_{0}=10\times \frac{k_{0}-4.3}{7.9-4.3}$ ms. The generated post-synaptic spikes *z*_*t*_ = 1 were then classified into three groups corresponding to the three species. Fifteen randomly chosen data points were assigned as the test set; the remainder were used as the training dataset. The other conditions were those described in section 3.2. The results are summarized in Figure 4. The average classification accuracy over 100 trials was 89.5 ± 5.3% for the training dataset and 89.5 ± 7.5% for the test dataset.

For comparison, this subsection introduces the results of the *multilayer ReSuMe* algorithm (Sporea and Grüning, 2013), a supervised learning algorithm for multilayer SNNs in rate coding. The multilayer ReSuMe algorithm was also evaluated on the iris flower dataset. In this evaluation, a trial was deemed successful if the classification accuracy exceeded 95% on the training dataset. After weeding out the unsuccessful trials, the proposed learning algorithm and multilayer ReSuMe algorithm achieved a classification accuracy of 94.7 ± 6.5 and 94.0 ± 0.79% respectively, on the test dataset (see Table 3). Despite its single-layer architecture, the SNN trained by the proposed learning algorithm classified the iris flower dataset at least as accurately as the multilayer SNN trained by the multilayer ReSuMe algorithm.

**Table 3:** Classification accuracies in the iris flower dataset after selection.

**Model**	**Unsupervised**		**Supervised**	
	**Training**	**Test**	**Training**	**Test**
MB model^*^	96.1 ± 0.7	94.7 ± 6.5	96.7 ± 1.1	92.2 ± 7.4
BB model^**^	96.2 ± 0.5	93.3 ± 5.4	96.7 ± 0.9	94.2 ± 5.8
Multilayer ReSuMe^***^	–	–	96.0 ± 0.0	94.0 ± 0.8

* *Proposed SNN based on the Multinoulli-Bernoulli model (section 3.3)*.

** *Proposed SNN based on the Bernoulli–Bernoulli model (section 3.7)*.

*** *Sporea and Grüning, 2013*.

### 3.4. Results for the MNIST dataset

**Figure 5:**
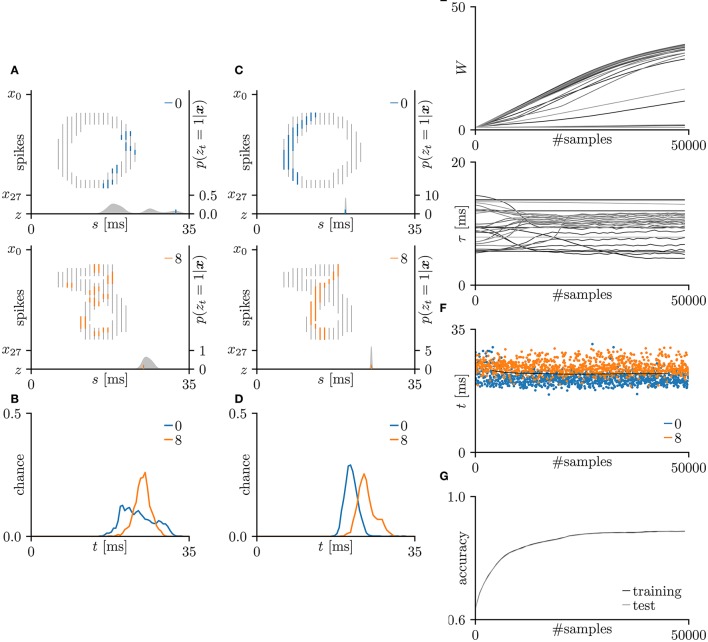
Results of SNN based on the Multinoulli-Bernoulli mixture model, evaluated on the hand-written digits 0 and 8 in the MNIST dataset (LeCun et al., 1998). **(A)** Representative spatio-temporal spike patterns of digit 0 (top panel) and digit 8 (bottom panel), and the post-synaptic spikes *z*_*t*_ = 1 before learning. Thick colored lines indicate the pre-synaptic spikes *x*_*is*_ = 1, elicited within μ − 1 < Δ*t* = *s* + τ_*i*_ − *t* < μ + 1 for *z*_*t*_ = 1; other pre-synaptic spikes *x*_*is*_ are depicted as thin gray lines (the left vertical axes). Gray shaded areas denote the probability of eliciting a post-synaptic spikes *z*_*t*_ = 1 per 1 ms (the right vertical axes). **(B)** Test distributions of the post-synaptic spike timings *z*_*t*_ = 1 before learning. **(C)** Post-synaptic spike timings *z*_*t*_ = 1 after learning, given the spatio-temporal spike patterns ***x*** in **(A)**. **(D)** Test distributions of the post-synaptic spike timing *z*_*t*_ = 1 after learning. **(E)** Trajectories of the synaptic weights *W*_*i*_ and conduction delays τ_*i*_ during the learning procedure. The darkest lines denote the values of the parameters *W*_0_ and τ_0_, and the lighter lines denote *W*_*i*_ and τ_*i*_ for *i* = 1, 2, … . **(F)** Random samples of the post-synaptic spike timings *z*_*t*_ = 1. The black solid line marks the decision boundary between spike patterns the hand-written digits 0 and 8. **(G)** Classification accuracy in the training and test datasets, each averaged over 100 trials.

**Figure 6:**
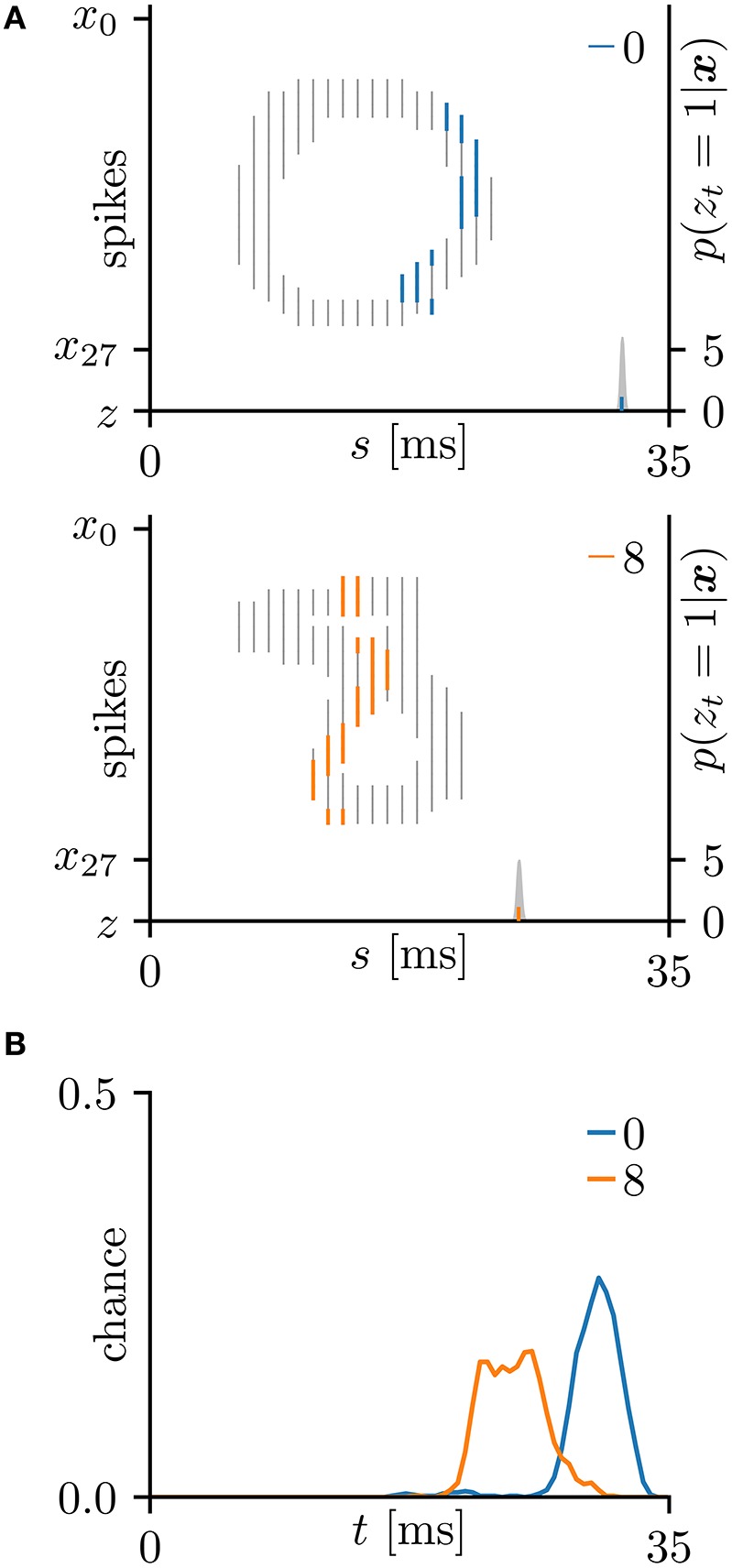
Other results on the hand-written digits 0 and 8 in the MNIST dataset (LeCun et al., 1998). **(A)** Representative spatio-temporal spike patterns of digit 0 (top panel) and digit 8 (bottom panel), and the post-synaptic spikes *z*_*t*_ = 1 after the learning procedure as per Figure 5C. Thick colored lines indicate the pre-synaptic spikes *x*_*is*_ = 1, elicited within μ − 1 < Δ*t* = *s* + τ_*i*_ − *t* < μ+1 for *z*_*t*_ = 1; other pre-synaptic spikes *x*_*is*_ are depicted as thin gray lines (the left vertical axes). Gray shaded areas denote the probability of eliciting a post-synaptic spikes *z*_*t*_ = 1 per 1 ms (the right vertical axes). **(B)** Test distributions of the post-synaptic spike timing *z*_*t*_ = 1 after learning. The SNN selectively responds to the reversed C-shaped edges, so the post-synaptic spikes *z*_*t*_ = 1 responding to “8” are earlier than those responding to “0” (in contrast to Figure 5).

In this subsection, the proposed learning algorithm was evaluated on the MNIST dataset (LeCun et al., 1998), which contains 28 × 28 grayscale images of 70,000 handwritten digits. In standard rate coding, the pixel intensities are represented by 28 × 28 = 784 pre-synaptic neurons (Nessler et al., 2009; Beyeler et al., 2013; O'Connor et al., 2013; Querlioz et al., 2013; Neftci et al., 2014; Diehl and Cook, 2015; Zambrano and Bohte, 2016). To emphasize the temporal coding, this study instead represented the rows and columns of the images by 28 pre-synaptic neurons and their 28 corresponding spike timings, respectively. When a pre-synaptic neuron *x*_*i*_ generated a spike at time *s*, the intensity of the pixel in row *i* and column *s* of the image was considered to exceed 0.5 (see Figure 5A). For simplicity, only the digits 0 and 8 were included in the analysis. After removing the other digits, 13,728 images were available. This experiment employed a 10-fold cross-validation: 10 % of images were randomly chosen for the test set. The results are summarized in Figure 5. This experiment yielded much more pre-synaptic spikes *x*_*is*_ = 1 than the previous two experiments. During transmission, the pre-synaptic spikes *x*_*is*_ = 1 that largely contributed to the post-synaptic spike *z*_*t*_ = 1 were emphasized. More specifically, if μ−1 < Δ*t* = *s*+τ_*i*_−*t* < μ+1 for the post-synaptic spike *z*_*t*_ = 1, the pre-synaptic spikes *x*_*is*_ = 1 was emphasized with a thick color line, whereas other pre-synaptic spikes *x*_*is*_ = 1 were attenuated (see Figures 5A,C). According to Figure 5C, the C-shaped edges are heightened, indicating that the SNN selectively detects and responds to these edges. In general, the C-shaped edge appears on the left side of digit 0 and in the center of digit 8, so the post-synaptic spikes *z*_*t*_ = 1 responded earlier to “0” than to “8”. In some trials, the SNN responded to reversed C-shaped edges (or to edges in digits such as “7”), and the post-synaptic spikes *z*_*t*_ = 1 responded earlier to “8” than to “0” (see Figure 6). The average classification accuracy over 100 trials was 88.7 ± 3.4% for the training dataset and 88.7 ± 3.7% for the test dataset.

### 3.5. Results without delay learning

The proposed learning algorithm was also evaluated without delay learning. More specifically, the synaptic weights *W*_*i*_ were updated by Equation (5), and the conduction delays τ_*i*_ were clamped to their initial values (i.e., were not updated by Equation 4). The other conditions were those described in previous subsections. All of the accuracies were drastically reduced (see Table 4).

**Table 4:** Classification accuracy of the proposed SNN without delay learning.

**Dataset**	**Unsupervised (Fixed delays)**	
	**Training**	**Test**
Toy spike patterns	94.9 ± 0.6	94.4 ± 0.9
Iris flower dataset	78.5 ± 12.0	77.2 ± 15.0
MNIST dataset	72.9 ± 11.8	72.9 ± 11.8

### 3.6. Results with supervised learning

The proposed learning algorithm is adaptable to supervised learning, in which synaptic plasticity and delay learning are not self-driven by generated post-synaptic spikes, but are driven by external spikes ${\tilde{z}}_{t}=1$ generated at the desired timings (Bohte et al., 2002; Ponulak, 2005; Gütig and Sompolinsky, 2006; Pfister et al., 2006; Paugam-Moisy et al., 2008; Taherkhani et al., 2015; Matsubara and Torikai, 2016). However, the desired timings ${\tilde{z}}_{t}=1$ are generally unknown. In this study, the spatio-temporal spike patterns ***x*** are classified by whether the post-synaptic spike timing *z*_*t*_ = 1 arrives earlier or later than the boundary decision. The group with the latest average post-synaptic spike timing should generate the most delayed post-synaptic spike. Therefore, the “late” group were given an external post-synaptic spike ${\tilde{z}}_{t+d}=1$ (where *d* > 0) after the generated post-synaptic spike *z*_*t*_ = 1. Meanwhile, the “early” group were given a post-synaptic spike ${\tilde{z}}_{t-d}=1$ before the generated post-synaptic spike *z*_*t*_ = 1. These post-synaptic spikes *z*_*t*+*d*_ = 1 and *z*_*t*−*d*_ = 1 tend to delay or advance the generated post-synaptic spike *z*_*t*_ = 1, respectively. In this study, the time parameter *d* was set to δ = 0.05 ms. The results of supervised learning are summarized in Tables 2, 3. In almost every case, the classification accuracy was improved, confirming that the proposed learning algorithm can be adapted to supervised learning without manually adjusting the spike timings.

### 3.7. Results with multiple post-synaptic spikes

**Figure 7:**
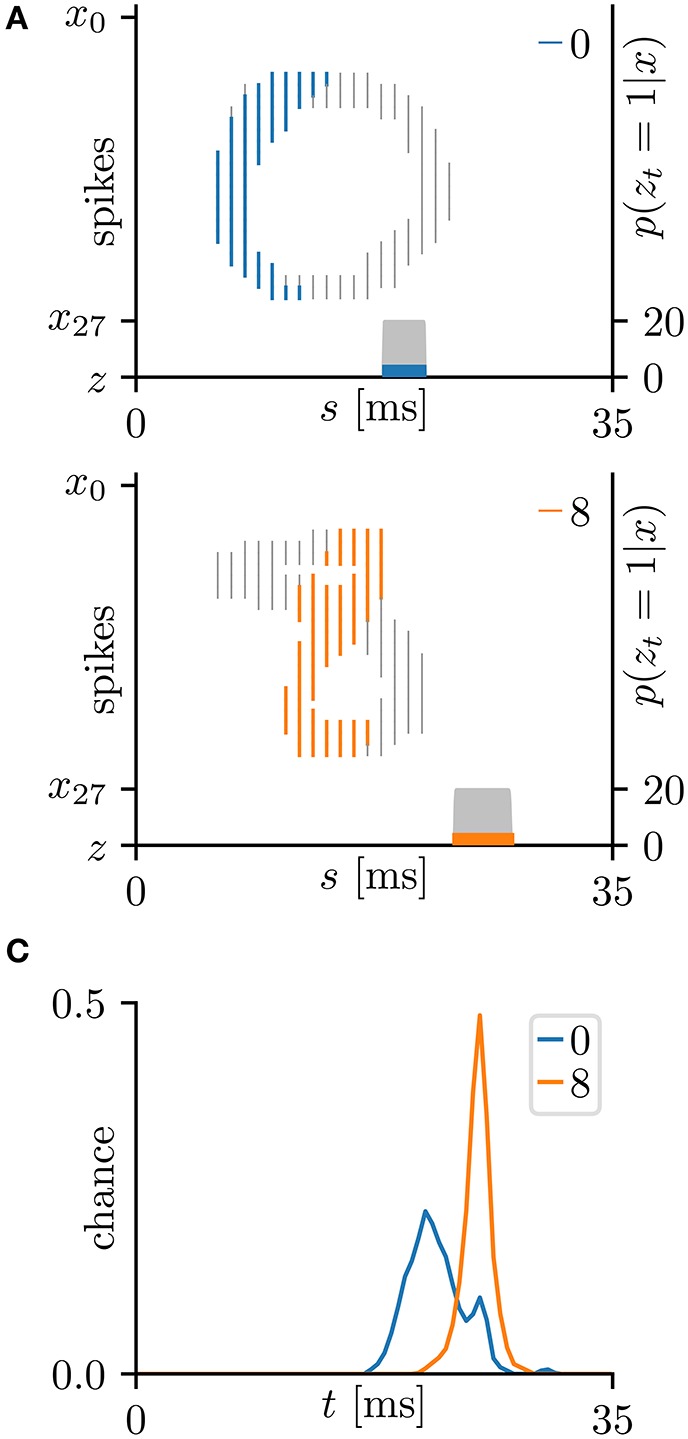
Results of SNN based on the Bernoulli–Bernoulli mixture model for the hand-written digits 0 and 8 in the MNIST dataset (LeCun et al., 1998). **(A)** Representative spatio-temporal spike patterns of digit 0 (top panel) and digit 8 (bottom panel), and the post-synaptic spikes *z*_*t*_ = 1 after the learning procedure as per Figure 5C. Thick colored lines indicate the pre-synaptic spikes *x*_*is*_ = 1, elicited within μ − 1 < Δ*t* = *s* + τ_*i*_ − *t* < μ+1 for *z*_*t*_ = 1; other pre-synaptic spikes *x*_*is*_ are depicted as thin gray lines (the left vertical axes). Gray shaded areas denote the probability of eliciting a post-synaptic spikes *z*_*t*_ = 1 per 1 ms (the right vertical axes). **(B)** Test distribution of mean timings of the post-synaptic spikes *z*_*t*_ = 1 after learning. Like the Multinoulli-Bernoulli mixture model (see Figure 5), the SNN selectively responds to the C-shaped edges.

This subsection evaluates the SNN based on the Bernoulli–Bernoulli mixture model described section 2.5. The homeostatic plasticity parameters were set to *b*_+_ = 0.01 and *b*_−_ = 0.0001, indicating that, on average, one in every hundred spatio-temporal spike patterns induces no post-synaptic spike *z*_*t*_ = 1. To determine the decision boundaries, the absence of the post-synaptic spike *z*_*t*_ = 1 was considered to indicate an exceptionally late post-synaptic spike (e.g., *z*_*T*_ = 1). As shown in Tables 3, 5, the classification accuracy was improved in the iris flower dataset but degraded in the MNIST dataset. As an example, Figure 7 shows the results of applying multiple post-synaptic spikes in the MNIST dataset.

**Table 5:** Classification accuracy of the proposed SNN based on thle Bernoulli–Bernoulli mixture model.

**Dataset**	**Unsupervised**		**Supervised**	
	**Training**	**Test**	**Training**	**Test**
Toy spike patterns	98.9 ± 3.0	98.9 ± 2.8	99.3 ± 2.3	98.1 ± 5.7
Iris flower dataset	90.1 ± 5.1	90.7 ± 7.8	93.1 ± 4.7	90.8 ± 7.7
MNIST dataset	83.7 ± 1.2	83.3 ± 1.5	84.8 ± 1.7	84.4 ± 1.8

## 4. Discussion

Rate-coding SNNs have already succeeded in various unsupervised and supervised learning tasks (Brader et al., 2007; Nessler et al., 2009; Beyeler et al., 2013; O'Connor et al., 2013; Diehl and Cook, 2015; Zambrano and Bohte, 2016), and have provided the opportunity for efficient computational architectures (Querlioz et al., 2013; Neftci et al., 2014; Cao et al., 2015). However, rate coding requires repeated sampling of the generated spikes, which increases the computational time (VanRullen and Thorpe, 2001). The alternative approach is temporal coding, which encodes the information into a spatio-temporal spike pattern. Temporal coding requires the timing of at least one spike (Bohte et al., 2002; Ponulak, 2005; Gütig and Sompolinsky, 2006; Pfister et al., 2006; Paugam-Moisy et al., 2008; Yu et al., 2014; Taherkhani et al., 2015), potentially enabling more efficient computational architectures than those based on rate coding (Matsubara and Torikai, 2013, 2016). These studies computed gradients of spike timing or excitatory post-synaptic potential with respect to synaptic weight and/or conduction delay, and adjusted to them to elicit post-synaptic spikes at the desired timings. However, they focused exclusively on supervised learning algorithms, which always require teacher spikes at the desired timings. In general, the desired timings are unknown and require careful manual adjustment. Therefore, this approach limits the flexibility in the context of machine learning and poorly represents biological systems that self-adapt to changing environments. Unsupervised delay learning algorithms have received far less attention than supervised learning. The few studies published in this area have not approached practical tasks such as classification and reproduction of given spike patterns (Hüning et al., 1998; Eurich et al., 1999, 2000). In contrast, the proposed unsupervised learning algorithm approximates the EM algorithm (see section 2), and can therefore classify the given spatio-temporal spike patterns by inferring their hidden causes. Under the proposed learning algorithm, the SNN also detected frequent patterns in the given spatio-temporal spike patterns. In addition, the proposed learning algorithm can be adapted to supervised learning without the application of externally determined spike timings.

The proposed learning algorithm relates the synaptic weight modification Δ*W* to the temporal difference Δ*t* and the current synaptic weight *W* (see section 3.1). When the temporal difference Δ*t* is positive, the synapse is potentiated; when Δ*t* is negative or largely positive, it is depressed. This relationship is consistent with previous electrophysiological studies (Markram et al., 1997; Bi and Poo, 1998) and has been discussed in theoretical studies (Shouval et al., 2002; Wittenberg and Wang, 2006; Shouval et al., 2010). The amount of synaptic modification Δ*W*_*i*_ decreases with increasing current synaptic weight *W*_*i*_. This relationship is also consistent with previous electrophysiological study (Bi and Poo, 1998) and is considered to maintain neuronal activity by suppressing excessive potentiation (Van Rossum et al., 2000; Gütig et al., 2003; Shouval et al., 2010; Matsubara and Uehara, 2016). The Results of the iris flower dataset (summarized in Figure 4) demonstrate that during the early learning phase, the pre-synaptic spikes are not simultaneously delivered to the post-synaptic neuron, and the synaptic weights and conduction delays change only gradually. After 50,000 samples, the conduction delays rapidly changed and almost converged to certain values before 60,000 samples. At that time, the pre-synaptic spikes arrived at the post-synaptic neuron simultaneously. Following the changing conduction delays, the synaptic weights increase, post-synaptic spikes become clustered, and the classification accuracy becomes higher (see Figures 4E–G). Similar time courses were observed for the toy spike patterns (see Figure 3). These results support Fields (2015) and Baraban et al. (2016), who hypothesized that conduction delay is adjusted for the synchronous arrival of multiple pre-synaptic spikes before the synaptic modification dominates. They also demonstrated that clustered post-synaptic spikes and accurate classification require the combined optimization of conduction delay and synaptic weight. Before the synaptic weights increase, the classification accuracy is poor. Therefore, delay learning and synaptic plasticity are inherently linked as mentioned by Jamann et al. (2017). Although, the detailed of biological delay learning (i.e., activity-dependent myelination) remains unclear, this study provides a good hypothetical explanation of the mechanism underlying this process.

With the MNIST dataset, the test accuracies of the proposed algorithm based on the Multinoulli-Bernoulli mixture model were almost equal to the corresponding train accuracies as summarized in Tables 2, 4. This could be because the MNIST has a limited number of local minima insensitively to the separation of the test dataset. Since the experiment employed a 10-fold cross-validation, the average test accuracy becomes equal to the average train accuracy if the proposed algorithm always converges to the same local minimum. Actually, in all the 100 trials, the proposed algorithm apparently converged to one of two local minima shown in Figures 5, 6.

When the conduction delays were fixed, the classification accuracy of the proposed learning algorithm drastically reduced (see Table 4). This occurred because the classification depends on the timing *t* of the post-synaptic spike *z*_*t*_ = 1, which remained almost static the delay modification. This result demonstrates one advantage of the proposed learning algorithm over other temporal coding learning algorithm such as the ReSuMe algorithm (Ponulak, 2005; Sporea and Grüning, 2013) and the Tempotron algorithm (Gütig and Sompolinsky, 2006; Yu et al., 2014), which learn by synaptic modification only. Gerstner et al. (1996) and Bohte et al. (2002) assumed multiple paths from a single source to a post-synaptic neuron with various delays. In such a situation, synaptic modification can adjust the post-synaptic spike timing by pruning the inappropriate paths leaving the appropriate ones only. However, an SNN based on this approach either requires numerous unused paths for future development, or its flexibility is limited by the initial network connections and conduction delays. SNNs optimized by delay learning algorithms such as the proposed learning algorithm are more efficient and flexible. A performance comparison between the two learning approaches is outside the scope of this paper, but is a worthwhile future task.

Despite the single-layer architecture, the classification accuracy of the SNN trained by the proposed learning algorithm is comparable to (even slightly superior to) that of multilayer ReSuMe, a supervised learning algorithm designed for multilayer SNNs (Sporea and Grüning, 2013). Supervised learning algorithms such as variants of ReSuMe (Ponulak, 2005; Sporea and Grüning, 2013; Taherkhani et al., 2015) and Tempotron (Gütig and Sompolinsky, 2006; Yu et al., 2014) require the desired timings of post-synaptic spikes, which must be determined before the learning procedure. As the desired timings are generally unknown, they should be adjusted manually and carefully in classification tasks. On the other hand, the proposed learning algorithm automatically determines the desired timing from the given dataset and the initial parameter values, even during a supervised learning. The removal of the need for external timing is another advantage of the proposed learning algorithm.

In temporal coding, the proposed learning algorithm requires fewer parameters and fewer spikes than existing learning algorithms in rate coding. Single-layer SNN in rate coding required 4 pre-synaptic and 3 post-synaptic neurons with 15 parameters (4 weight parameters and a bias parameter for each group) for the iris flower dataset, and more than 784 neurons and parameters for 2 digits in the MNIST dataset (e.g., Nessler et al., 2009; Neftci et al., 2014). In these algorithms, each pre-synaptic neuron corresponds to a feature or a pixel, and each post-synaptic neuron corresponds to a group. As a single neuron represents multiple values by its spike timing in temporal coding, the proposed neural network requires 4 pre-synaptic neurons and 1 post-synaptic neuron with 10 parameters (4 synaptic weights *W*_*i*_, 4 conduction delays τ_*i*_, and 2 decision boundaries) for the iris flower dataset, and only 29 neurons and 57 parameters for 2 digits in the MNIST dataset. In addition, each feature is the iris flower dataset was encoded into a single spike timing with in the range 0–10 ms with a time step of δ = 0.05 ms. Therefore, the resolution of each feature was 200 stages. To achieve the same resolution in rate coding, the pre-synaptic spikes must be generated up to 200 times. The proposed learning algorithm requires far fewer pre-synaptic spikes. In future works, rate coding in the proposed and existing learning algorithms should be compared with similar numbers of parameters and spikes. Note that, as the proposed SNNs have only one post-synaptic neuron, they can classify two or three groups at most. To classify more groups, the proposed SNN must be generalized to multiple interacting post-synaptic neurons.

This study proposed two types of SNNs; one based on the Multinoulli-Bernoulli mixture model, the other on the Bernoulli–Bernoulli mixture model. The former takes a single post-synaptic spike from the temporal distribution, whereas the latter can independently elicit a post-synaptic spike at every time step and exhibits a burst-like behavior. The SNN based on the Bernoulli–Bernoulli mixture model classified the iris flower dataset more accurately, and the MNIST dataset less accurately, than the SNN based on the Multinoulli-Bernoulli mixture model. Owing to the multiple generations of post-synaptic spikes, there was little variation among trials in the iris flower dataset, and the learning proceeded more robustly than in the SNN based on the Multinoulli-Bernoulli mixture model. Consequently, the accuracy was improved in this dataset. Conversely, the number of pre-synaptic spikes representing a single image varied widely in the MNIST dataset, generating a widely varying number of post-synaptic spikes. The SNN based on the Bernoulli–Bernoulli mixture model sometimes detected too many edges similar to the template edge in various sub-regions, and sometimes detected no edge. Meanwhile, the SNN based on the Multinoulli-Bernoulli mixture model always detected the single edge that best fitted the template edge. Therefore, the learning of the SNN based on the Bernoulli–Bernoulli mixture model proceeded less robustly, lowering the classification accuracy of the MNIST dataset.

The SNN proposed in section 2.1 can also be implemented in continuous time. However, the number of pre-synaptic spikes *x*_*is*_ = 1 is unlimited in this mode, and the joint probability *p*(***x***, ***z***) of the Multinoulli-Bernoulli and Bernoulli–Bernoulli mixture models is difficult to define. For this reason, the SNN was implemented in discrete time. The EPSP of the proposed SNN is the linear sum of the EPSPs induced by multiple pre-synaptic spikes *x*_*is*_ = 1, and the proposed learning algorithms in sections 2.3 and 2.5 were normalized by the time step δ. Hence, the proposed SNN is robust to the time step δ and can be approximated to continuous time as the time step δ approaches 0.

Note that Equation (4) and the first term in Equation (5) are independent of the time step δ but the second term in Equation (5) does depend on δ. When adjusting the time step δ,

In contrast, the behavior of dynamical spiking neurons described by an ordinary differential equation is sensitive to the time step and the numerical simulation algorithm (Hansel et al., 1998). A continuous-time SNN and its corresponding generative model will be explored in future work.

The Bernoulli–Bernoulli mixture model independently draws a post-synaptic spike *z*_*t*_ = 1 at each time step. However, biological studies have confirmed that the intervals between the generated spikes do not follow a Poisson distribution, implying that the spikes in biological neural networks are not independently generated (Burns and Webb, 1976; Levine, 1991). When a dynamical neuron generates a spike, it enters the relative refractory period. During this time, the neuron becomes less sensitive to stimuli and is less likely to generate a spike. After the relative refractory period, the neuron returns to its resting state and resumes its usual chance of generating a spike (Izhikevich, 2006a, Chapter 8). Incorporated with these dynamics, the SNN based on the Bernoulli–Bernoulli mixture model would be purged of its burst-like behavior in the MNIST dataset (which degraded the classification accuracy), and would be rendered more computationally efficient and more biologically realistic. A dynamic version of the proposed SNN is a further opportunity for future work.

## 5. Conclusion

This study proposed an unsupervised learning algorithm that adjusts the conduction delays and synaptic weights in an SNN. The proposed learning algorithm approximates the Expectation-Maximization (EM) algorithm and was confirmed to classify the spatio-temporal spike patterns in several practical problems. In addition, the proposed learning algorithm is adjustable to supervised learning, which improves its classification accuracy. The formulation of the proposed learning algorithm is partially consistent with the synaptic plasticity demonstrated in previous biological and theoretical studies. Therefore, the proposed learning algorithm is a strong candidate model of biological delay learning and will contribute to further investigations of SNNs in temporal coding.

## Author contributions

The author confirms being the sole contributor of this work and approved it for publication.

### Conflict of interest statement

The author declares that the research was conducted in the absence of any commercial or financial relationships that could be construed as a potential conflict of interest.
